# Sugarcane Pulp Take-Out Containers Produce More Microparticles in Acidic Foods

**DOI:** 10.3390/foods12132496

**Published:** 2023-06-27

**Authors:** Yi Hu, Chun-Ru Mo, Zhi-Wei Wang, Wen-Wen Yu, Chang-Ying Hu

**Affiliations:** 1 Institute of Packaging Engineering, Jinan University, Zhuhai 519070, China; huyi999@stu2020.jnu.edu.cn (Y.H.);; 2 Department of Food Science & Engineering, Jinan University, Huangpu West Avenue 601, Guangzhou 510632, China; mochunru@stu2021.jnu.edu.cn

**Keywords:** sugarcane pulp take-out container, microparticles, quantitative analysis, particle size, food safety

## Abstract

In the current study, the production of microparticles released from fifteen commercial sugarcane pulp (SCP) take-out containers into different food simulants under different conditions was investigated, where deionized water (DI water), 4% acetic acid (4% HAc), and 95% ethanol (95% EtOH) were used to simulate aqueous, acidic, and fatty foods, respectively. Results showed that compared with DI water and 95% EtOH, 4% HAc caused the degradation of sugarcane fibers, thereby releasing the highest number of microparticles. The overall migration values of the sugarcane pulp take-out containers in 4% HAc were above the prescribed limit of 10 mg/dm^2^. Furthermore, it was estimated that consumers may intake 36,400–231,700 microparticles in a take-out container at one time, of which the proportion of particles with a particle size between 10 and 500 μm was the highest, ranging from 26,470 to 216,060 items. Moreover, the Al and Fe are the main metals in these take-out containers, ranging between 35.16 and 1244.04 and 44.71 and 398.52 mg/kg, respectively, followed by Pb, Ti, and Sr. This study provides important information that the safety of both the production of microparticles and the metallic elements should be considered for SCP take-out containers when in contact with food.

## 1. Introduction

Along with the rapid development of the internet and e-commerce markets, the number of online orders has dramatically increased following the consumption of disposable take-out containers [1]. In 2022, there were over 60 million disposable take-out containers discarded each day [2], which has inevitably posed challenges to the entire public health system and social environment all over the world [2,3]. There is an urgent need to replace plastic food packaging materials (i.e., disposable take-out containers) using biodegradable, compostable, and environmentally friendly materials such as biodegradable plant resource components or wastes [4,5,6].

Sugarcane pulp (SCP) is a compostable environmentally friendly material made from residual bagasse from the sugar industry through homogenizing, molding, shaping, and disinfection. The expanded utilization of SCP in making biodegradable food packing materials relieves the huge pressure on the environment brought by disposable non-degradable take-out containers, while it also enables the utilization of agricultural wastes. In general, SCP contains 33.5–55% glucan, 17–32% hemicellulose, 17–32% lignin, and 0.7–8% ash [7,8]. In 2020, global sugarcane cultivation was 187 million tons [9], most of which was used for papermaking. SCP disposable take-out containers, which have the characteristics of lightness and moderate strength and toughness and are biodegradable and oil/water resistance, have been widely used in Eastern and Western countries for food packaging [10,11].

Theoretically, the heavy metal content in food packaging materials made from plant-based wastes is generally high [12,13,14,15]; Ranjan et al. [16] and Liu et al. [17] found that the release of microplastics into hot water may also increase the concentration of heavy metals, which could pose a health hazard. In addition to (heavy) metals, the released microplastics (particle size < 5 mm) are also a cause for concern [16,18]. For example, studies have reported that microplastics can induce male reproductive toxicity and intestinal microflora imbalance and inflammation in mice [19,20], while the microplastics released from polypropylene (PP) (particle diameter < 20 μm) can cause cytotoxicity by increasing reactive oxygen species [21]. Accordingly, similar to PP/polystyrene (PS) take-out containers, it is reasonable to assume that SCP take-out containers may also produce microparticles when in contact with foods [22]. Fadare et al. [18] determined the averaged weight of the isolated microparticles to be as high as 38 ± 5.29 mg per plastic container, with various morphological characteristics. Du et al. [22] speculated that microplastic intake through containers may be up to 203 items/person/week. At present, however, there are only few studies investigating the release of microparticles from food contact materials (FCMs), especially from sugarcane pulp take-out containers.

Accordingly, in the current study, fifteen brands of commercial SCP take-out containers were purchased in China. Fourier Transform-Infrared Spectrometry (FTIR) and Inductively Coupled Plasma-Mass Spectrometry (ICP-MS) were used to identify the functional groups and to measure the content of (heavy) metals within the take-out containers, respectively. Moreover, as real food ingredients are generally complex and it is often impractical to perform migration and specification tests on real foods under real conditions, it was therefore necessary to use simplified food models (i.e., food simulators), which can almost truly reflect the migration of components from food contact materials or products to the foods they come into contact with, to conduct the migration experiments, as stated in GB 31604.1-2015 [23]. Consequently, DI water, 4% HAc, and 95% EtOH were used as aqueous, acidic, and fatty foods simulants, respectively, to analyze the overall migration as well as the migration of microparticles under different conditions (i.e., temperature and time). Lastly, laser diffraction (LSD) and scanning electron microscopy (SEM) were used for the size distribution analysis and quantitative analysis of microparticles. This is the first study to systematically investigate how different foods, including aqueous, acidic, and fatty foods, influence the release of microparticles under different conditions. This study will provide scientific suggestions and opinions to SCP take-out container manufacturers and consumers.

## 2. Materials and Methods

### 2.1. Chemicals

Ethanol, acetic acid, and nitric acid were of analytical–reagent grade from Macklin Biochemical Co., Ltd. (Shanghai, China). Water was prepared by a ultrapure water system (Milli-Q, Millipore, MA, USA).

### 2.2. Collection of SCP Take-Out Containers

In total, fifteen types/brands of SCP take-out containers from nine provinces in China were purchased in June 2021. The purchased take-out containers, referred to as S-1 to S-15, respectively, (Table 1) were sealed and stored in a dark place. While most take-out containers can withstand temperatures as high as 100 °C, the maximum temperature for S-13 was only 80 °C.

### 2.3. Overall Migration

Overall migration refers to the total amount of non-volatile material transferred from food contact material to a specific analog solvent at a specific temperature and time. It is usually expressed as milligrams of non-volatile migrations per kilogram of the food simulators (mg/kg), or milligrams of non-volatile migrations per square decimeter of contact area (mg/dm^2^). As all SCP take-out containers were purchased from different companies and showed differences in terms of the density and thickness, the immersing area of each take-out container was therefore used.

Referring to the regulations in GB 31604.1-2015 [23] and in combination with the expected use of the take-out container, the overall migration test conditions were set at 70 °C for 2 h. Food simulants including deionized water (DI water), 4% acetic acid (4% HAc), and 95% ethanol (95% EtOH) were used to represent real aqueous, acidic, and fatty foods so as to eliminate the influence of impurities in real foods. Other test conditions were carried out according to GB5009.156-2016 [24] and GB 31604.8-2016 [25].

Namely, for each take-out container, six pieces of fragments (each with a surface area of 1 cm^2^) were strictly selected and then immersed with 20 mL food simulants (1 kg/L), to give a volume/contact area ratio of 6 dm^2^/kg. After immersing with food simulants at 70 °C for 2 h, the solution was collected, transferred to a pre-dried glass evaporating dish, and placed in a water bath (HH-4, Juchuang, Qingdao, China) to evaporate for 30–60 min. The mixture was dried at 105 ± 2 °C in an oven for another 2 h. After cooling to room temperature in a desiccator, the dish was weighted. For the overall migration tests in contact with all three food simulants, the overall migration value was calculated using Equation (1):

(1) $$X_{i}=\frac{\left(M_{1}-M_{2}\right)}{V_{2}\times S}\times V_{1}$$

Here, *X_i_* is the overall migration value of the SCP take-out container, mg/dm^2^; *M*_1_ is the residue weight of the immersing solution, mg; *M*_2_ is the residual mass of the blank (only the food simulants without immersing with the take-out container pieces), mg; *V*_1_ is the total volume of soaking solution, mL; *V*_2_ is the evaporated volume of soaking solution, mL. In the current study, *V*_1_ = *V*_2_; *S* represented the area of contact with the take-out container, namely, 12 cm^2^.

### 2.4. Collection of the Evaporation Residue Particles (ERPs)

As stated in Section 2.3, for all take-out containers, the residues after the overall migration tests when in contact with all three types of food simulants were collected, mixed, and freeze-dried. The freeze-dried residues were called evaporation residue particles (ERPs).

### 2.5. Cluster Analysis

Based on the overall migration results described in Section 2.3, cluster analysis was carried out with the overall migration value of each take-out container into different food simulants settings as variables, the inter-group connection as the clustering method, and the Euclidean distance as the measurement interval. The aim was to classify all fifteen take-out containers into different groups based on the overall migration results into different food simulants. Based on the cluster analysis results, representative take-out containers were then selected for the following analysis of the microparticles.

### 2.6. Fourier Transform Infrared Spectroscopy (FTIR) Analysis

Fourier Transform Infrared Spectroscopy (FTIR) (Nicolet iS50 + iN10, Thermo Fisher Scientific, Waltham, MA, USA) was used to identify the chemical groups of all SCP take-out containers and the evaporation residue particles (ERPs).

Prior to analysis, all SCP take-out containers, except ERPs, which were already fine powders, were shredded/cut into small pieces, frozen with liquid nitrogen, and then ground into fine powders. The transmission method, with a spectral range of 4000–400 cm^−1^ and 64 scans, was used.

### 2.7. Inductively Coupled Plasma-Mass Spectrometry Analysis (ICP-MS)

The concentrations of metallic elements of take-out containers S-1 to S-15 were measured using ICP-MS (iCAP RQ, ThermoFisher Scientific Inc., Waltham, MA, USA) according to the method of Xie et al. [26] with minor modifications. In brief, 0.5 g of each SCP powder was weighted and mixed with 50 mL nitric acid for digestion. The digestion solution was then diluted tenfold for analysis by ICP-MS. For ERPs, only 0.05 g ERPs was weighted and mixed with 10 mL nitric acid for digestion and then analyzed using the ICP-MS. Here, only one parallel was executed.

### 2.8. Collection of Microparticles

For collecting the microparticles released from the take-out containers when in contact with food simulants, the total volume of food simulant was used based on the maximum capacity of the take-out container as specified by the manufacturer (Table 1). During the experiment, it was observed that when in contact with 95% EtOH, the liquid leaked significantly (Figure S1). Accordingly, for all food simulants including DI water, 4% HAc, and 95% EtOH, the full immersion method was adopted. That is, each take-out container was cut into equal quarters and then completely immersed with the food simulants, which had been preheated to 70 °C. The solution containing released microparticles after immersing with the food simulants under different temperatures (70 or 100 °C) or times (30 or 120 min), respectively, were obtained and filtered using a vacuum pump (SHZ-DIII, Yuhua Instrument Co., Ltd., Gongyi, China) and sand core filter device (T-50, Jinteng experimental equipment Co., Ltd., Tianjin, China) combined with a nylon filter membrane (0.22 μm, Jinteng experimental equipment Co., Ltd., Tianjin, China). For the solution immersed with 95% EtOH, a polytetrafluoron filter membrane (0.22 μm, Jinteng experimental equipment Co., Ltd., Tianjin, China) instead of a nylon filter membrane was used. The microparticles that remained on the filter paper were rinsed into a glass triangular conical bottle using exactly 15 mL DI water.

For the washed solution containing microparticles, 1 mL was stored at −20 °C, which was further used to conduct quantitative and morphological analysis of the microparticles. The remaining solution was then used for diameter or particle size analysis. In order to avoid contamination from the environment, the laboratory doors and windows were closed during the whole experimental process, and three sets of parallel and blank controls were set.

### 2.9. Measurement of the Particle Diameters

The particle diameters of the collected microparticles were measured immediately after extraction using the LSD particle diameter distribution apparatus (SALD-2300, Shimadzu, Kyoto, Japan). For the Shimadzu SALD-2300, the LSD scattering method was applied to measure the particle diameters of the collected microparticles released after immersing with food simulants (0.17–2500 μm). The machine automatically adjusted the refractive index so that the diameter of the microparticles could be measured properly. Preliminary experiments showed that when the refractive index was 1.35-0.00 i, the particle diameter was in the range of 1–1000 μm. In order to reduce accidental errors (i.e., the anisotropy of particle distribution), triplicate measurements were set in parallel, and the averaged value was recorded.

### 2.10. Quantitative Analysis and Morphological Observation of the Microparticles

The morphology and the number of collected microparticles were visualized using SEM (EVO MA15, Carl Zeiss AG, Oberkochen, Germany).

In brief, 10 μL solution containing microparticles as prepared in Section 2.8 was dropped onto a copper platform covered with Al conductive adhesive, dried in an oven at 30 °C, and coated with a gold coating for visualization. A fixed and appropriate magnification was chosen so that all microparticles could be captured in one graph. Based on SEM images, the total number of microparticles was calculated manually with the help of counting tools in Adobe Photoshop. For each sample, three determinations were performed, and the final value was the average of the three determinations. The conversion between the quantity and concentration of microparticles released by the take-out container when immersed with food simulants was as follows:

(2) $$C=\frac{Q_{i}\times 15\times 1000}{V_{0}}$$

Here, *C* represents the total concentration of microparticles in the solution released from the take-out container when immersing with different food simulants (items/mL); *Q_i_* is the averaged value of the total number of microparticles observed in SEM image with triplicates; 15 is the total volume of the washed liquid, mL; *V*_0_ is the volume of food simulants used for immersion, mL; the value 100 is the computational multiple of 10 μL to 1 mL.

### 2.11. Data Analysis

Microsoft Excel 2019 was used for data processing and image rendering. Cluster analysis and significance analysis were performed using IBM SPSS Statistics 25. A Kruskal-Wallis one-way ANOVA test was used to analyze the significance of the average migration value and 90D of take-out containers in different simulants (*p* < 0.05). Adobe Photoshop 2020 was used to conduct quantitative and morphological analysis.

## 3. Results and Discussion

### 3.1. Characterization of the Take-Out Containers

**Figure 1:**
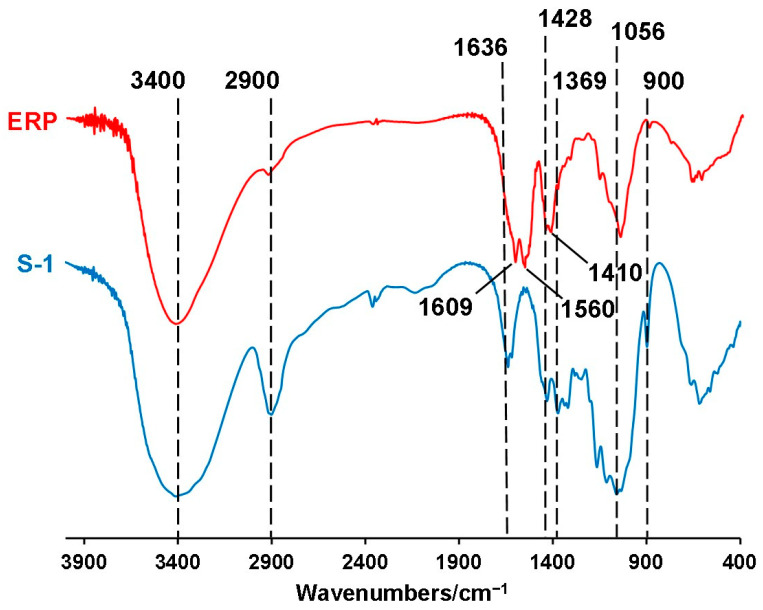
FITR results of take-out container made from sugarcane pulp (SCP) using S-1 as an example. The evaporation residue particles (ERPs) were the collection of all residues of all take-out containers (with three duplicities for each) to the three food simulants including DI water, 4% HAc, and 95% EtOH.

Figure 1 shows the typical FTIR spectra of SCP take-out containers using S-1 as an example, while that of all rest of the take-out containers is provided in Figure S2. For all SCP take-out containers, the infrared spectra were all similar to previous studies [11,27]. The peak at ~3400 cm^−1^ was mainly attributed to the vibration of hydrogen bonds in cellulose and hemicellulose hydroxyl (-OH) groups, and the peak near 2900 cm^−1^ was caused by the asymmetric stretching vibration of C-H groups in lignin and/or cellulose of the sugarcane fibers.

Compared to that of the take-out container, certain differences were observed in the FTIR peaks of ERPs. Note that ERPs were collected as a mixture of all the migrated components from the take-out container to the food simulants (DI water, 4% HAc, and 95% EtOH) at 70 °C for 2 h. Compared with S-1, the peaks of ERPs at ~2900 cm^−1^ and 900 cm^−1^ almost disappeared, representing the stretching vibration of hydroxyl groups (-OH) in cellulose/hemicellulose and C-O-H/C-O-C of sugar rings in hemicellulose and lignin. This indicated that only trace amounts of cellulose/lignin migrated from SCP disposable take-out containers when immersed with these food simulants at 70 °C for 2 h. Of the ERPs, the peak at ~1636 cm^−1^ also disappeared, whereas the new peak at ~1609 cm^−1^ was probably caused by the vibration of the carboxyl group (-COO) in 4% HAc remaining in ERP, as seen in a previous study as well [28]. Moreover, of ERPs, the peaks at ~1428 and ~1369 cm^−1^, respectively, were probably created by the vibrations of aliphatic or aromatic C–H and C–O groups in lignins [27,28]. The peaks ranging between 1369 and ~1316 cm^−1^ were mainly attributed to the C-O and/or O-H vibrations of hemicellulose and/or lignin groups and may also have been caused by the bending vibration of C-O and C-H bonds in the aromatic ring.

### 3.2. Metal Content of SCP Take-Out Containers

When grown in contaminated soil, plants may accumulate metals, which may eventually pass into the processed products (i.e., tableware and take-out container). As shown in Table 2, of all SCP take-out containers except S-11, Al and Fe were the main metals, ranging from 35.16 to 1244.04 and from 44.71 to 398.52 mg per kg take-out container, respectively, followed by Pb, Ti, and Sr. For Al, as no such high concentration was generally detected in either sugarcane fibers or its relevant products, it thus can be speculated that the high content of Al in SCP disposable take-out containers might be introduced because of the addition of additives (i.e., aluminate coupling agent or titanium dioxide, which are generally used as surfactants for disposable take-out containers) [29,30].

Sugarcane plants may also contain a relatively high content of Fe, Zn, Sr, Mn, and other micronutrients such as Ce and Ba. Interestingly, unlike other take-out containers, the content of Pb in S-11 was the highest, namely, 136 mg per kg take-out container (Table 2). This result has also been observed previously [31]. During plant growth, Pb may be accumulated in the roots [32]. The large amount of Pb in S-11 may have been either related to the soil or industrial emissions [33]. This suggests that attention should be paid to heavy metal element contamination of take-out containers made from plant wastes (i.e., wheat or sugarcane straw) so that their content can be kept in a safe range.

Lastly, the averaged concentration of metallic elements of all take-out containers was also calculated and was found in the order of Al > Fe > Pb > Ti > Sr > Mn > Zn > Ba > Zr > Cr. As for the metal element content in ERPs, it was found that the averaged content of metal elements was significantly higher than that in the original take-out container counterparts. For example, the average content of Al in ERPs was 21,779.86 mg per kg take-out container, which was almost 100 times higher than that of the original take-out container counterparts. This was probably because when immersing with food simulants, the metal elements more easily migrated into the food simulants, and the adsorption of ERPs further increased their contents in ERPs [17].

### 3.3. Overall Migration of SCP Take-Out Containers

**Figure 2:**
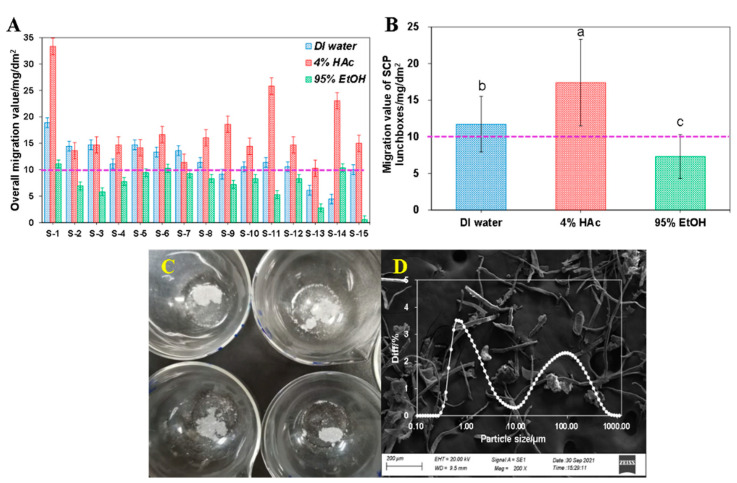
Individual (**A**) and averaged (**B**) overall migration value of all take-out container (*n* = 3) into different food simulants including DI water, 4% HAc, and 95% EtOH. (**C**) The evaporation residues observed in the experiment. (**D**) The SEM graph and the particle size distribution of the ERP. Different letters indicate significant differences at *p* < 0.05 (*n* = 15).

Table 3 and Figure 2A show the overall migration results of the SCP take-out containers after immersion with different food simulants at 70 °C for 2 h. As is shown clearly, compared with either DI water or 95% EtOH, the total weight of migrating components from the take-out container to 4% HAc food simulant was the highest. For example, when immersing with 4% HAc, the overall migration of all take-out containers ranged between 33.33 ± 1.18 mg/dm^2^ for S-1 and 10.28 ± 0.39 mg/dm^2^ for S-13, respectively. When immersing with DI water, the overall migration value was between 4.44 ± 0.39 and 14.72 ± 1.42 mg/dm^2^, whereas it ranged between 0.56 ± 0.39 and 11.11 ± 1.04 mg/dm^2^ when immersing with 95% EtOH. In fact, there are rich acidic Chinese dishes, including sour soup with fat beef, Guizhou sour soup, etc., and also the common sour dessert, sour plum soup. Although they are less acidic than 4% HAc, there is indeed a greater risk of serving these foods in take-out containers compared to other types of foods.

As shown in Figure 2B, significant differences were observed in terms of the averaged migration values of all take-out containers in contact with different food simulants. In detail, after immersing with 4% HAc at 70 °C for 2 h, it was found that the averaged overall migration of all fifteen take-out containers was 17.41 mg/dm^2^, followed by that with DI water and then with 95% EtOH, with an average number of 11.72 and 7.31 mg/dm^2^, respectively. This was probably because under acidic conditions, the lignocellulosic (i.e., cellulose and lignin) in the SCP take-out containers degraded and leached out [34]. Moreover, although compared with DI water, the 95% EtOH food simulant has a stronger penetration ability and can quickly penetrate into the take-out containers, nevertheless, in this study, our results showed that the overall migration value of take-out containers when immersed with DI water was higher than that in the 95% EtOH food simulant (Figure 2B). This may have been caused by the filler or surface coating added to the take-out containers. Lastly, as shown in Figure 2C,D, after freezer-drying, the white flocculent precipitates (ERPs) were mainly composed of fibers with different lengths, while some spherical particles with much smaller sizes also existed. The diameters of the ERPs were found to be in the range of 0.1–1000 μm, which is in the category of microparticles (particle diameter < 5 mm) [18,20,22].

### 3.4. Cluster Analysis

**Figure 3:**
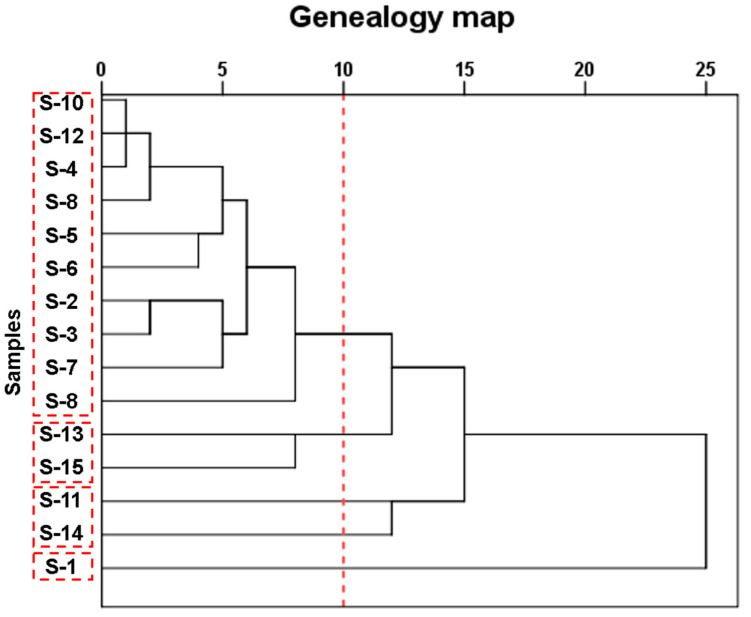
Cluster analysis results.

In order to explore how immersion temperature, immersion time, and the type of food simulants, including DI water, 4% HAc, and 95% EtOH, impact the diameter of the released microparticles from the SCP take-out containers, a fast and simple systematic clustering analysis was conducted using the overall migration value to each food simulant as variables. The cross-section method is usually used to observe the number of classifications, that is, a line is used to cut off the classification line to obtain the classification result. The horizontal axis of the graph shows the relative distances of each class. The smaller the value is, the more the clustering number is, and the fuzzier the class features are. As shown in Figure 3, all fifteen take-out containers could be classified into four classes. In detail, while S-1 performed completely differently from the rest of the take-out containers, S-11 and S-14 performed similarly and thus could be clustered together. Moreover, while both S-13 and S-15 performed similarly, the remaining types of take-out containers all performed similarly to each other and thus were grouped together.

### 3.5. Impacts of Immersing Conditions on the Production of Microparticles

#### 3.5.1. 90D

**Figure 4:**
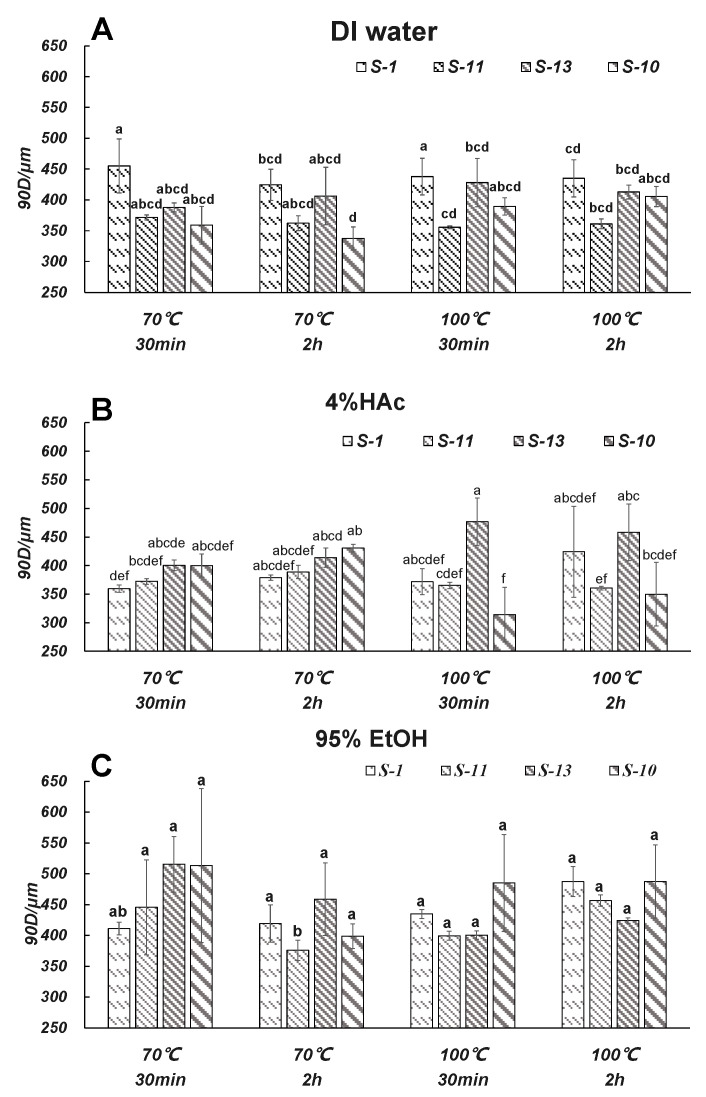
Particle sizes of microparticles of the four take-out containers when in contact with DI water (**A**), 4% HAc (**B**), and 95% EtOH (**C**) under different conditions. Letters above data bars indicate statistical differences in concentration among samples (*p* < 0.05, *n* = 3).

Figure 4 shows the particle sizes of the microparticles of the four brands of commercial SCP take-out containers after immersion with different food simulants at two temperatures (70 and 100 °C, respectively) and immersing times (30 and 120 min, respectively). D90 is the particle size corresponding to a sample when the cumulative particle size distribution number reaches 90%. For example, when immersing with DI water at 70 °C for either 30 or 120 min, the 90D of the macroparticle produced by S-13 was significantly smaller than the remaining three brands of take-out containers, especially S-1. Nevertheless, when immersing with the 4% HAc food simulant, the 90D of the microparticles produced by S-13 was significantly larger than that of the S-1. This thus necessitated future research to be conducted to investigate how the interaction between the SCP take-out container and food simulant affects the migration properties of the microparticles.

When the immersing temperature increased from 70 to 100 °C, it was found that the particle diameter of the microparticles generated by S-13 showed fewer differences with other take-out containers. According to the manufacturer’s instructions, the S-13 is not suitable for temperatures of 80 °C and above (Table 1). This suggests that the changes in the temperature of the packaged food could significantly influence the diameter of the released microparticles. This is different from a previous study where the author reported that for plastic disposable take-out containers (i.e., PP/PS), the treatment of hot water or shaking showed no significant influence on the microplastic abundance of take-out containers. This is probably because the disposable take-out container made from plant wastes is more vulnerable to the temperature of the packaged food.

Now when we compared the diameters (90D) of the microparticles produced by S-13 when immersed with DI water and 4% HAc at 70 °C, respectively, it was found that the particle diameters of the microparticles produced by 4% HAc were significantly larger than those produced by DI water. This suggests that the nature of the packaged foods (i.e., water-soluble food or oily food) leads to significant variations in the particle size of microparticles released from the take-out containers. Compared with water-soluble foods, it was less safe when packing acidic foods.

#### 3.5.2. Particle Size Distributions of Microparticles in Contact with Different Food Simulants

**Figure 5:**
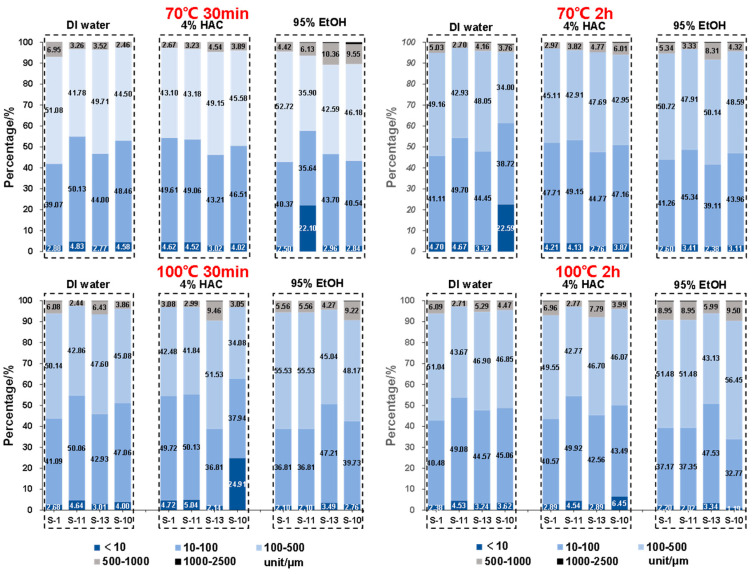
The percentage of microparticles released by different take-out containers in contact with different food simulants including DI water, 4% HAc, and 95% EtOH. The whole range of microparticles was sub-divided into five fractions based on its averaged particle diameters, including Fra I (<10 μm), Fra II (10–100 μm), Fra III (100–500 μm), Fra IV (500–100 μm), and Fra V (1000–2500 μm). The value was based on triplicate measurements.

Based on the cumulative distribution data of the microparticles released from different take-out containers when in contact with different food simulants under different conditions, the whole range of microparticles was sub-divided into five fractions, including <10, 10–100, 100–500, 500–1000, and 1000–2500 μm (Fra I–V). In the first place, as shown in Figure 5, under different conditions (i.e., immersing time and temperature), significant differences were observed. It was found that for all take-out containers, almost no microparticles with particle sizes > 1000 μm were released, whereas the largest proportion of microparticles ranged between 10 and 500 μm, including Fra II and Fra III. The sizes of microparticles generated from sugarcane pulp take-out containers were much larger than the maximum particle size detected in plastic material, i.e., 31.7 μm in disposable plastic materials [17].

(1) DI water

When in contact with DI water at 70 °C for 30 min, of all SCP take-out containers, the percentage of Fra I (<10 μm) ranged between 2.88 and 4.58% and between 4.70 and 22.58% when the immersing time increased from 30 to 120 min. As noted, for S-10, when immersing with the DI water at 70 °C for 120 min, the percentage of the microparticles with the particle size < 10 μm (Fra I) reached 22.58%, which was significantly higher than that of the rest of the take-out containers. If we increased the temperature to 100 °C, the percentage of the microparticles with particle sizes < 10 μm (Fra I) was between 2.68 and 4.64% and 2.38 and 4.53% when immersing for 30 and 120 min, respectively. These results suggest that with increased temperature, the content of Fra I did not necessarily increase accordingly.

(2) 4% HAc

When in contact with 4% HAc at 70 °C for 30 min, of all the SCP take-out containers, the percentage of Fra I (<10 μm) was between 3.02 and 4.62%, and the percentage was between 43.21 and 49.61% for Fra II (10–100 μm), 43.10 and 49.15% for Fra III (100–500 μm), and 2.67 and 4.54% for Fra IV (500–1000 μm), respectively. Except for S-10, for the remaining three take-out containers, no significant differences were observed, even though the immersion time and temperature were changed. Particularly, for S-10, when the temperature increased from 70 to 100 °C, the percentage of microparticles with particle size < 10 μm (Fra I) increased dramatically from 4.02% to 24.91%. However, at 100 °C, when the immersion time increased from 30 to 120 min, the percentage of the released microparticles with particle sizes < 10 μm (Fra I) decreased significantly, namely, from 24.91% to 6.45%. This suggests that the conditions, especially the temperature of the food simulant, significantly influenced the particle size distribution of the microparticles, while it is also easily understood that different take-out containers purchased from different company performed differently.

(3) 95% EtOH

Compared with DI water and 4% HAc, oily foods are more frequently consumed in Asian countries, especially in China, which is generally represented by 95% EtOH. As shown in Figure 5, when immersing with 95% EtOH at 70 °C for 30 min, the particle size distribution of the microparticles released from the four take-out containers ranged between 2.50 and 22.10% (Fra I, <10 μm), 35.64 and 43.70% (Fra II, 10–100 μm), 35.90 and 52.72% (Fra III, 100–500 μm), and 4.42 and 10.35% (Fra IV, 500–1000 μm), respectively. When the temperature reached 100 °C, the particle size distribution of each fraction was in the range of 2.1–3.49% (Fra I), 36.81–47.21% (Fra II), 45.04–55.53% (Fra III), and 4.27–9.22% (Fra IV), respectively.

### 3.6. Quantitative Analysis of Microparticles

**Figure 6:**
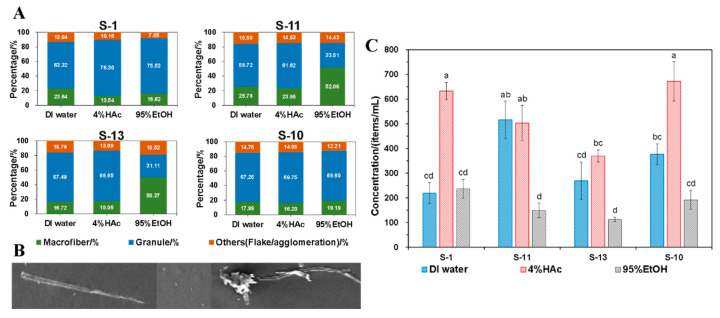
(**A**) Quantitative analysis of microparticles released from take-out containers to different food simulants including DI water, 4% HAc, and 95% EtOH. Each type of take-out container included three replicates (*n* = 3). (**B**) SEM observation of the microparticles. (**C**) Representation of the total items of microparticles when in contact with different food simulants of each type of commercial take-out container. Each take-out container included three replicates. Different letters indicate significant differences at *p* < 0.05 (*n* = 3).

As stated in Section 2.10, SEM was adopted to observe the influence of different food simulants on the release of microparticles quantitatively. To do this, the immersion temperature was fixed at 70 °C with a total immersing time of 120 min. Moreover, as shown in Figure 6B, when calculating the total items of microparticles, the long fiber particles observed were regarded as microfiber particles, while the spherical and punctate particles presented were regarded as granules, as marked in Figure 6. The interlacing, twining, and lamellar particles were all recorded as other categories.

As shown in Figure 6A, S-1 produced more fibrous particles when in contact with DI water, while S-11 and S-13 released more fibrous particles when in contact with 95% EtOH, accounting for more than 50% of the total number of microparticles. On the contrary, S-10 showed certain differences, that is, no significant differences in the proportion of microparticles with different morphological characters were observed when in contact with different food simulants. Similar to the overall migration results, the total number of microparticles (item/mL food simulant) produced by immersion with different simulants was the largest in 4% HAc, followed by DI water and 95% EtOH (Figure 6C). Moreover, although actions have been taken to avoid the impact of the environment on the results, a lower content of microparticles was still detected in the blank sample and thus needed to be deleted. After deducting the blank, results showed that there was still a high concentration of microparticles, and the total number of microparticles released by the SCP take-out container into different food simulants was in the range of 104–662 items/mL food and was 52–331 items/mL food when translated into one-sided contact.

### 3.7. Estimation of Microparticle from SCP Take-Out Container by Humans

There has also been much research of microplastics in polypropylene feeding bottles [35], tea bags [36], paper cups [16], and plastic take-out containers [17,18,22]. Although sugarcane pulp take-out containers are environmentally friendly and biodegradable, the amount of microparticles produced by them cannot be ignored. In this study, human intake of microparticles via the SCP take-out container has also been calculated based on the average abundance of microparticles in SCP containers. As mentioned above, the average number of microparticles released by the SCP take-out container into food simulants was in the range of 52–331 items/mL food. Based on the overall migration results and the assumption that the internal surface of the SCP take-out container with a volume of 700 mL is 9 dm^2^ when in contact with food or food simulants, it can be estimated that the consumer may intake 5.04–299.97 mg or 36,400–231,700 items of microparticles at one time along with the diet. This was far higher than that reported by Du, namely, 203 items/person/week, by using plastic containers [22]. Since the filter membrane we used had a pore size of 0.2 microns, the microparticles we evaluated were all on the micron scale.

In combination with the particle size distributions of the microplastics produced from the take-out containers when immersed with food simulants at 70 °C and 2 h (Figure 5), the intake of the microparticles with different particle sizes can therefore be estimated, including the maximum content and number of microparticles with different sizes. In detail, the consumer may intake 866–52,341 items of microparticles with diameter < 10 μm, 14,094–115,155 items of microplastics ranging between 10 and 100 μm, 12,376–117,518 items between 100 and 500 μm, and 983–19,254 items between 500 and 1000 μm.

## 4. Conclusions

In this study, the production of microparticles from SCP take-out containers when in contact with different food simulants including DI water, 4% HAc, and 95% EtOH under different times and temperatures was investigated. Our results showed that compared with DI water and 95% EtOH, the 4% HAc, which is usually used to represent acidic foods, might cause degradation of the take-out containers, resulting in the highest number of microparticles, which is also significantly greater than the specified limit value. This suggests that when packaging acid food, the safety of the SCP take-out container should be considered, especially in terms of the released microparticles. Moreover, the consumer may ingest 36,400–231,700 items per meal, of which the proportion of microparticles with particle size between 10 and 500 μm is the largest, with a number of 26,470–216,060 items. The accumulation of metal elements or organic matter carried by microparticles should not be ignored either.

The sugarcane pulp take-out container has developed rapidly in recent years due to its superior functions and environmental protection characteristics. This study shows that acidic food has a certain negative impact on its safety, including the release of microparticles and heavy metals. Therefore, it is necessary to further regulate the market of SCP disposable take-out containers and supervise its quality. In addition, how to reduce the microparticles produced by degradable take-out containers made from plant wastes and the toxicity of such microparticles still need further research.

## Figures and Tables

**Table 1:** Basic information about disposable sugarcane pulp (SCP) take-out containers.

No.	Sample ID	Volume (mL)	Resources	Thickness (mm)	Water/ Oil Proof	Microwave/ Freezing
1	S-1	700	Guangzhou, China	0.73 ± 0.02	Yes	Yes
2	S-2	700	Suzhou, China	0.72 ± 0.03		
3	S-3	500	Shandong, China	0.69 ± 0.00		
4	S-4	450	Sichuan, China	0.60 ± 0.03		
5	S-5	550	Zhejiang, China	0.74 ± 0.02		
6	S-6	450	Zhejiang, China	0.63 ± 0.05		
7	S-7	500	Shanghai, China	0.63 ± 0.02		
8	S-8	500	Anhui, China	0.66 ± 0.02		
9	S-9	600	Shanghai, China	0.65 ± 0.02		
10	S-10	450	Shanghai, China	0.50 ± 0.01		
11	S-11	650	Shanghai, China	0.61 ± 0.03		
12	S-12	500	Jiangsu, China	0.62 ± 0.03		
13	S-13	600	Fujian, China	0.56 ± 0.08		
14	S-14	500	Tianjin, China	0.71 ± 0.02		
15	S-15	500	Zhejiang, China	0.75 ± 0.07		

**Table 2:** Content of different metal elements in sugarcane pulp (SCP) take-out containers.

mg/kg	Al	Fe	Pb	Ti	Sr	Mn	Zn	Ba	Cr	Cu
LOQ	2.00	0.10	0.05	0.05	0.09	0.03	0.10	0.05	0.04	0.02
S-1	1244.04	398.52	2.60	15.05	7.25	13.01	12.23	3.32	4.70	1.01
S-2	133.26	44.71	0.56	3.89	5.13	2.57	3.30	1.64	-	-
S-3	35.16	50.78	0.61	3.29	6.66	1.78	1.29	0.63	2.16	0.15
S-4	91.74	135.84	1.96	9.58	5.81	2.90	8.00	1.50	-	0.39
S-5	195.24	160.26	0.60	7.94	2.71	5.49	2.30	1.32	0.93	-
S-6	46.00	169.84	0.27	6.44	4.60	3.00	-	0.68	0.97	-
S-7	37.38	75.32	0.09	5.17	5.59	2.37	0.71	1.41	-	-
S-8	152.89	96.58	0.24	7.69	2.44	5.55	-	1.41	-	-
S-9	105.02	177.52	0.44	3.62	5.11	3.62	0.58	2.12	-	0.27
S-10	37.44	94.42	0.54	6.06	3.75	1.94	0.29	3.37	-	0.29
S-11	38.11	48.62	136.43	5.13	4.30	2.03	0.31	1.80	-	-
S-12	74.49	86.43	0.67	6.85	4.74	4.86	-	1.01	0.80	-
S-13	48.34	127.29	1.25	10.16	2.51	2.55	3.78	1.21	-	0.98
S-14	650.85	54.00	1.71	3.29	2.74	1.79	0.66	0.66	0.52	-
S-15	541.97	69.54	0.20	7.93	3.03	2.71	0.99	0.95	1.57	0.71
Averaged	228.80	119.31	9.88	6.81	4.42	3.74	2.30	1.54	0.78	0.25
ERP	21,779.86	3320.18	57.98	189.87	478.50	363.19	508.72	129.10	54.31	23.04

Averaged: the averaged concentration of the metal element of all fifteen types of SCP take-out containers. “-”: Not detected.

**Table 3:** Overall migration value of sugarcane pulp (SCP) take-out containers when immersed with different food simulants at 70 °C for 2 h (mg/dm^2^) (*n* = 3).

Samples	Food Simulants		
	4% HAc	DI Water	95% EtOH
S-1	33.33 ± 1.18^a^	18.89 ± 2.19 ^b^	11.11 ± 1.04 ^c^
S-2	13.61 ± 2.19 ^a^	14.44 ± 1.71 ^a^	6.94 ± 0.79 ^b^
S-3	14.72 ± 0.79 ^a^	14.72 ± 1.42 ^a^	5.83 ± 0.68 ^b^
S-4	14.72 ± 0.79 ^a^	11.11 ± 1.42 ^b^	7.78 ± 0.39 ^b^
S-5	14.17 ± 0.68 ^a^	14.72 ± 1.42 ^a^	9.44 ± 1.71 ^b^
S-6	16.67 ± 1.80 ^a^	13.33 ± 1.80 ^ab^	10.28 ± 0.39 ^b^
S-7	11.39 ± 0.39 ^ab^	13.61 ± 1.57 ^a^	9.27 ± 2.58 ^b^
S-8	16.04 ± 1.42 ^a^	11.39 ± 0.39 ^b^	8.33 ± 1.36 ^b^
S-9	18.61 ± 1.04 ^a^	9.17 ± 2.04 ^b^	7.22 ± 1.04 ^b^
S-10	14.44 ± 0.79 ^a^	10.56 ± 0.39 ^b^	8.33 ± 1.36 ^c^
S-11	25.83 ± 1.36 ^a^	11.39 ± 0.39 ^b^	5.28 ± 0.79 ^c^
S-12	14.72 ± 1.42 ^a^	10.56 ± 0.79 ^b^	8.33 ± 1.36 ^b^
S-13	10.28 ± 0.39 a	6.11 ± 0.39 ^b^	2.78 ± 0.39 ^c^
S-14	23.06 ± 1.57 ^a^	4.44 ± 0.39 ^c^	10.38 ± 0.79 ^b^
S-15	15.00 ± 1.18 ^a^	10.00 ± 1.18 ^b^	0.56 ± 0.39 ^c^

Values in the same row with different superscripts are significantly different using one-way ANOVA tests (*p* < 0.05).

## Data Availability

The data used to support the findings of this study can be made available by the corresponding author upon request.

## Supplementary Materials

The following supporting information can be downloaded at: https://www.mdpi.com/article/10.3390/foods12132496/s1. Figure S1: The leaking phenomenon of take-out container when contacting with the 95% EtOH food simulant. Figure S2: Infrared spectra of 15 SCP take-out containers.
