# Supplementary material for: Sugarcane Pulp Take-Out Containers Produce More Microparticles in Acidic Foods
Source: Foods. 2023 Jun 27;12(13):2496. doi: 10.3390/foods12132496 (PMC10340730; doi:10.3390/foods12132496)
Supplement: Supplementary file 1 [file foods-12-02496-s001.zip › foods-2415407-supplementary.pdf]

Sugarcane pulp lunch-boxes produce more microparticles in acidic foods

Yi Hu[a], Chunru Mo[b], Zhiwei Wang[a], Wenwen Yu[b*], Changying Hu[a*]

[a] Packaging Engineering Institute, Jinan University, Zhuhai, Guangdong, 519070, China.

[b] Department of Food Science & Engineering, Jinan University, Huangpu West Avenue 601, Guangzhou City, 510632, Guangdong, China.

Corresponding authors at:

Changying Hu, hucy0000@sina.com

Address: Packaging Engineering Institute, Jinan University, Zhuhai, Guangdong, 519070, China.

Wenwen Yu, yuwenwen@*jnu*.edu.cn

Address: Department of Food Science & Engineering, Jinan University, Huangpu West Avenue 601, Guangzhou City, 510632, Guangdong, China.

Supplementary Information

Prepared for Submission
To *Foods*

Item	Description	Page
**Figure S1**	The leaking phenomenon of lunchbox when contacting with the 95% EtOH food simulant.	S3
**Figure S2**	Infrared spectra of 15 SCP lunchboxes.	S4

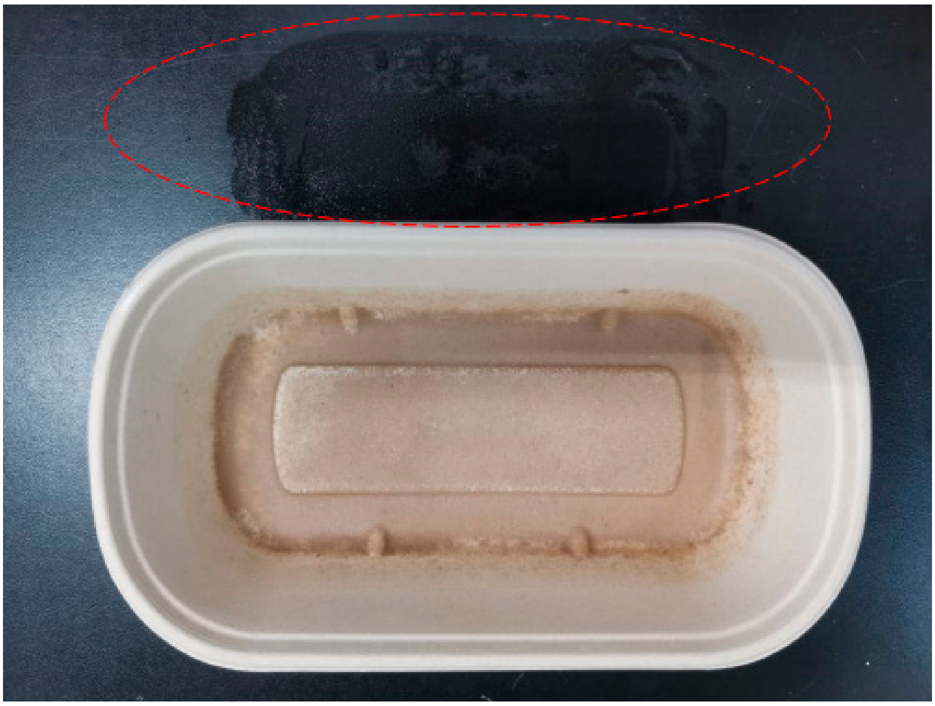

Figure S1. The leaking phenomenon of lunchbox when contacting with the 95% EtOH food simulant.

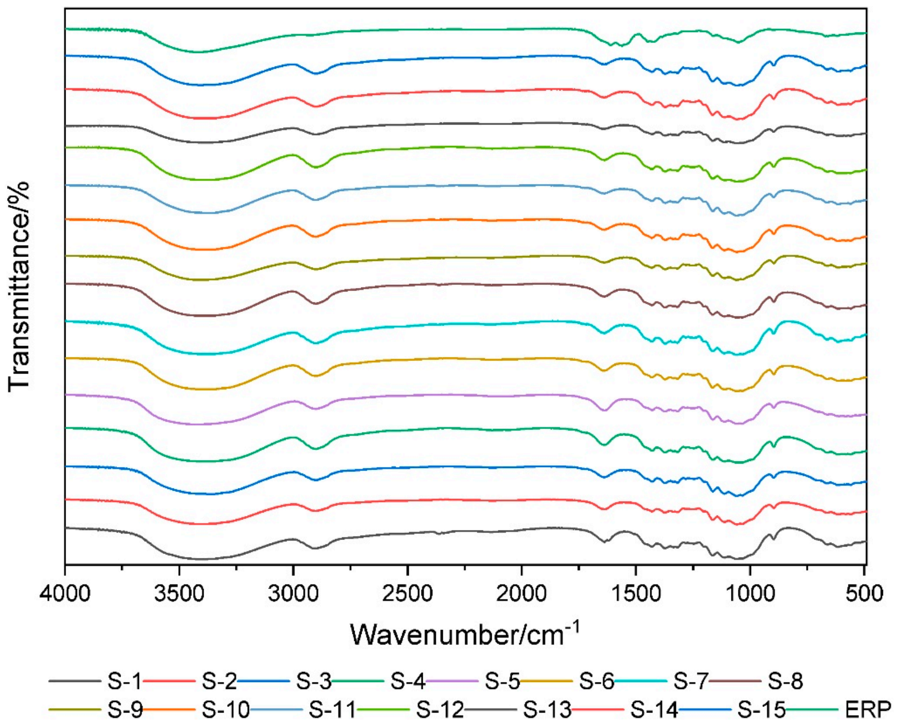

Figure S2. Infrared spectra of 15 SCP lunchboxes.
